# Details of exposure during childhood of the original children (G1s) in the ALSPAC study to aspects of religious faith and spirituality.

**DOI:** 10.12688/wellcomeopenres.25712.1

**Published:** 2026-02-03

**Authors:** Yasmin Iles-Caven, Steven Gregory, Hamid Reza Tohidinik, Jean Golding

**Affiliations:** 1 Centre for Academic Child Health, Population Health Sciences, University of Bristol Medical School, Bristol, England, BS8 2BN, UK

**Keywords:** ALSPAC; childhood; adolescence; faith; religiosity; spirituality; education; sex differences.

## Abstract

Information collected from the study parents on their own religious and spiritual beliefs and behaviours during the first 9 years following the birth of the ALSPAC study children has been documented previously in a Data Note. Here we describe the data collected on their *children’s* exposures to, and participation in, religious and/or spiritual events during their childhoods (defined as <25 years). Information available includes their exposures whether via school, places of worship or more informal gatherings. We describe the variables available and the frequencies of response and show the differences between the boys and girls (using sex ascribed at birth). Ways in which researchers can apply for appropriate data to analyse are described.

## Introduction

To assess possible influences on health and developmental outcomes of individuals it may be useful to consider their religious and/or spiritual beliefs and behaviours (RSBBs) whether as direct associations or as a mediator or moderator. There is increasing evidence that features of childhood are strongly related to adult mental health, but exposures to religion or spirituality during childhood or adolescence are rarely considered. Studies considering the positive or negative effects of religiosity have mainly started by obtaining information from adults, but few have looked at the longitudinal effects of exposure to aspects of RSBB during childhood. Consequently, this area is much under-researched.

Here we outline the relevant data that is available on the children taking part in the Avon Longitudinal Study of Parents and Children (ALSPAC) that can be linked to other social, psychological, environmental and health data of the individuals studied from early infancy through adolescence into early adulthood [ https://www.bristol.ac.uk/alspac/researchers/]. There is much discussion as to when childhood ends, but we have used the age of 25 since this is when the pre-frontal cortex of the brain finishes developing [ Arain *et al.*, 2013]. In addition, this age definition concurs with the definition used by the journal The Lancet Child and Adolescent Health [ https://www.thelancet.com/lanchi/about].

## Material and methods

### The ALSPAC cohorts

The Avon Longitudinal Study of Parents and Children (ALSPAC) started in 1990 with the aim of studying the ways in which the environment interacted with the genetic background to influence health and development [ Golding *et al.*, 2001]. Uniquely for the time, the study started during pregnancy and included information from both mothers and fathers [ Boyd *et al.*, 2013; Fraser *et al.*, 2013]. Among the environmental data collected during pregnancy from each parent were details of their own RSBBs [see Iles-Caven *et al.*, 2022 for details]. Data described in this Data Note were obtained using structured self-completed questionnaires sent to the mother (or chief carer) to report relevant data concerning the child and, after the age of 11, to the child participants themselves.

The study enrolled pregnant women who were resident in a defined area of Avon in south-west England and who had an expected date of delivery between 1 ^st^ April 1991 and 31 ^st^ December 1992 [ Boyd *et al.*, 2013; Fraser *et al.*, 2013]. Approximately 80% of the eligible population were enrolled at this time. Each pregnant woman was asked to invite her partner to take part, and she was sent a separate questionnaire to pass to him if he acquiesced [ Northstone *et al.*, 2023].

The initial number of pregnancies enrolled was 14,541. Of these initial pregnancies, a total of 14,676 fetuses, resulted in 14,062 live births and 13,988 children who were alive at 1 year of age. Starting when the oldest children were approximately 7 years of age, attempts were made to bolster the initial sample with eligible cases who had failed to join the study originally. As a result, when considering variables collected from the age of seven onwards data are available for more than the 14,541 pregnancies mentioned above. The number of new pregnancies not in the initial sample (known as Phase I enrolment) is 913. The phases of enrolment are described in more detail in the cohort profile paper and its update [ Northstone *et al.*, 2019]. The total sample size for analyses using any data collected after the age of seven is therefore 15,454 pregnancies, resulting in 15,658 fetuses. Of these, 14,901 children were alive at 1 year of age.

A large amount of information was collected on each of the parents (hereafter known as the G0s) as well as their offspring (the G1s). The study website contains details of all available data that is available through a fully searchable data dictionary and variable search tool. < https://www.bristol.ac.uk/alspac/researchers/our-data/>. PDF copies of the questionnaires are available, as are ‘built’ files, which contain the questions along with the frequencies of responses. From the age of 22, study data were collected and managed using REDCap electronic data capture tools hosted at the University of Bristol. REDCap (Research Electronic Data Capture) is a secure, web-based software platform designed to support data capture for research studies [ Harris *et al.*, 2009].

## Ethical approval

Ethical approval for the study was obtained from the ALSPAC Ethics and Law Committee and the Local Research Ethics Committees. Informed consent for use of all data collected was obtained from participants following the recommendations of the ALSPAC Ethics and Law Committee at the time. Participants can contact the study team at any time to retrospectively withdraw consent for their data to be used. Study participation is voluntary and during all data collection sweeps, information was provided on the intended use of data. The completion of questionnaires, either on paper or online, was considered to be written consent from participants to use their data for research purposes. For the majority of tests undertaken during face-to-face visits, verbal consent was obtained from participants (both parents and children as appropriate) prior to the start of any data collection. However, some tests required the completion of a written consent form. A full list of all ALSPAC ethical approvals can be found here: http://www.bristol.ac.uk/alspac/researchers/research-ethics/

### The questionnaires

Questions relating to religiosity or spirituality were mainly devised by the ALSPAC team with input from the ALSPAC Scientific Advisory Group as well as the ALSPAC Legal and Ethics Committee (ALEC). Advice was also obtained from a variety of specialists in the field. Each question was devised to be as unambiguous as possible and was formatted so that the respondents could mainly answer by ticking a box, but when requested, were also able to respond in text. (It should be noted that this part of the study was undertaken at a time when electronic questionnaires were rarely used with the general ALSPAC population.)

### Structure of this data note

Data provided in this publication include details of: (a) the actual question asked; (b) the frequencies of response including by sex, and (c) the relevant variable names. It should be noted that the frequencies may vary slightly over time due to the option that participants have of withdrawing their data at any time.

It should also be noted that this is a Data Note, which is not aimed to present detailed analyses, but rather as a description of all the relevant data available concerning the RSBB of the G1 children in the cohort.

## Results

### Child attendance at a faith group

**Figure 1.**
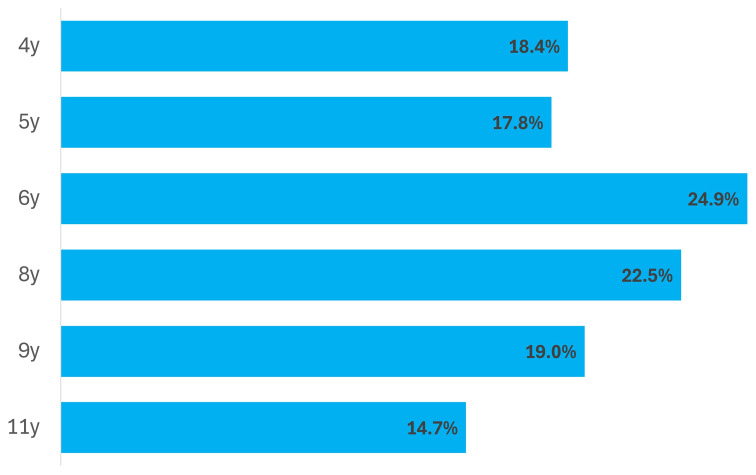
Proportion of ALSPAC children who attended Sunday school or other religious group by age.

Various questionnaires sent to the mother (or main carer) asked about the offspring’s attendance at Sunday school (a faith-based group which meets together to learn about religious subjects – often at the same time as their parents are attending a religious service). The questions asked are described in Table 1, and the frequencies of attendance of all children and of each sex separately in Table 2, Table 2a and Table 2b. Although there are some inconsistencies between the text of the questions asked, the proportion of all the children aged between 4 and 11 years who did attend Sunday School shows an interesting pattern ( Figure 1) with less than a fifth of children attending at each age before 6 years, a rise to a quarter at age 6 and a fall thereafter. This pattern, however, masked the fact that the boys and girls, when considered separately, showed a slight increase in those attending at least once a month between ages 4 and 5 years and a steady fall thereafter. At each age proportionately more girls than boys attended ( Table 2c).

**Table 1.** The exact wording of the questions asked about attendance at Sunday School and/or other faith-based groups during childhood.

Question asked	Age of child	Variable name(s)	No. children involved			M/F
			Total	Boys	Girls	
**About how often does he/she go to Sunday school?**	4 years	kl446	9434	4894	4540	1.08
**How often does he/she go to Sunday school or other religious group in term time?**	5 years	km4356	8905	4586	4319	1.06
**About how often does your child go to Sunday school**	6 years	kq563	8334	4292	4042	1.06
**About how often does he/she go to Sunday school?**	8 years	kt3005	8063	4114	3949	1.04
**How often does he/she go to Sunday school?**	9 years	ku524	8087	4097	3990	1.03
**How often does he/she go to Sunday school?**	11 years	kw9004	7244	3634	3610	1.01
**Does he/she attend a place of worship (church, mosque etc.)?**	9 years	ku416	7756	3910	3846	1.02
**Does he/she attend a place of worship (church, mosque, etc)?**	11 years	kw4125	7409	3705	3704	1.00
**In the last 4 weeks have you been to or used church/mosque/temple or other place of worship**	17 years	ccxd654	4500	1911	2589	0.74
**Have you used a Church/Mosque/ Temple/other place of worship in your local area in the last four weeks ^c^**	18+ years	cct1004	3125	1122	2003	0.56
**During this school year, have you taken part in any of the following activities? …Religious groups or organisations. ^d^**	School year 11 (16 yrs)	ccxa146	5435	2286	3149	0.73
**During this school year, have you taken part in any of the following activities? …Religious groups or organisations. ^e^**	School year 11 (16 yrs)	ccxa126	5435	2286	3149	0.73
**Did you ever participate in a faith-based group in childhood? ^f^**	NS	YPK1370	4030	1353	2677	0.51
**Did you ever participate in a faith-based group as a teenager? ^f^**	13–18 years	YPK1380	4030	1353	2677	0.51

M/F= Male/Female ratio; NS = not specified; RSBB = Religious and/or Spiritual Beliefs and Behaviours; ^a^questions asked of the mother (or main carer) about the child; ^b^questions asked of the child him/her-self; ^c^question asked online – actual question not available on built file. ^d^Responses for inside school, and ^e^outside school. ^f^exact wording is given in the text.

**Table 2.** Frequency of attendance of the children at Sunday school or other faith-based groups [n (%)] (see Table 1 for the actual wording of the questions asked and variable numbers).

Age (years)	1-6 times per week	Once per week	1-3 times per month	<once per month ^a^
4	24 (0.3)	1227 (13.0)	482 (5.1)	7711 (81.7)
5	70 (0.8)	1367 (15.3)	631 (7.1)	7111 (82.2)
6	30 (0.3)	1121 (13.4)	384 (4.6)	6809 (81.6)
8	37 (0.3)	1005 (12.5)	321 (4.0)	6718 (83.2)
9	19 (0.3)	784 (9.7)	346 (4.3)	6944 (85.8)
11	18 (0.2)	548 (7.6)	254 (3.5)	6432 (88.7)

^a^includes ‘not at all’

**Table 2a.** Frequency of *boys’* attendance at Sunday school or other faith-based group (see Table 1 for the actual wording of the questions asked and variable numbers).

Age (years)	1-6 times per week	Once per week	1-3 times per month	<once per month ^a^
4	16 (0.3)	608 (12.4)	241 (4.9)	4029 (82.3)
5	35 (0.8)	662 (14.4)	309 (6.7)	3580 (78.1)
6	13 (0.3)	524 (12.2)	183 (4.3)	3572 (83.2)
8	15 (0.4)	444 (10.8)	154 (3.7)	3501 (85.1)
9	7 (0.2)	356 (8.7)	151 (3.7)	3583 (87.5)
11	10 (0.3)	225 (6.2)	124 (3.4)	3275 (90.1)

^a^includes ‘not at all’

**Table 2b.** Frequency of *girls’* attendance at Sunday school or other faith-based group (see Table 1 for the actual wording of the questions asked and variable numbers). [N.B. frequency categories change slightly over time].

Age (years)	1-6 times per week	Once per week	1-3 times per month	<once per month ^a^
4	8 (0.2)	616 (13.6)	241 (5.1)	3672 (80.9)
5	35 (0.8)	702 (16.3)	322 (7.5)	3258 (75.4)
6	17 (0.4)	594 (14.7)	201 (5.0)	3230 (79.9)
8	12 (0.3)	561 (14.2)	163 (4.1)	3213 (81.4)
9	12 (0.3)	428 (10.7)	195 (4.9)	3355 (84.1)
11	8 (0.2)	323 (8.9)	130 (3.6)	3149 (87.2)

^a^includes ‘not at all’

**Table 2c.** Differences between the sexes in the proportion of children attending Sunday school or other faith-based group at least once a month.

Age (years)	Boys	Girls	Ratio M:F
4	17.6%	18.9%	0.93:1.00
5	21.9%	24.6%	0.89:1.00
6	16.8%	20.1%	0.84:1.00
8	14.9%	19.6%	0.76:1.00
9	12.6%	15.9%	0.79:1.00
11	9.9%	12.7%	0.78:1.00

Although no questions regarding faith during the teenage and early adult years were asked contemporaneously, when the G1s were aged 30 they were asked the following question: ‘at any time during your life have you participated in any of the following activities where you meet and interact with a regular group of other people? ….a faith-based group’. Possible responses included ‘yes in childhood’, ‘yes as a teenager’ and ‘no, not at all’. There were 4030 responses, which indicated that 20.1% of the G1s as children and 10.1% of teenagers had attended such a group. Despite, the retrospective nature of these figures, they are in reasonable accord with the data in Figure 1 if Sunday School is included as such a group and ‘childhood’ is assumed to be pre-puberty.

### Prayer

Unfortunately, ALSPAC did not collect information on whether the child was being taught to pray during the early years. By the age of 9 relatively few were noted to pray often by their mothers ( Table 3), with slightly more girls doing so than boys (12.7% v. 9.9%; Table 3a, Table 3b); although the proportion reported to pray often dropped between ages 9 and 11, the proportion of mothers reporting that they did not know increased. Nevertheless, at age 11 the proportion of boys who were reported to pray was lower than that of the girls ( Table 3a, Table 3b). It should be noted though that the mother was likely reporting prayer in the home rather than at a place of worship or at school.

**Table 3.** Frequency that child prays and/or attends a place of worship at ages 9 and 11 as reported by the mother/chief carer.

Age (years)	Variable	Activity	Often	Sometimes	Not at all	NK ^a^	N
9	ku417	Prays	11.2%	35.4%	44.6%	8.8%	7808
11	kw4126	Prays	8.1%	26.4%	48.9%	16.6%	7366
9	ku416	Attends ^b^	16.2%	29.6%	54.2%	-	7762
11	kw4125	Attends ^b^	13.5%	22.8%	63.8%	-	7417

^a^Not known; ^b^Attends a place of worship (church, mosque, etc.)

**Table 3a.** Frequency that *boys* pray and/or attend a place of worship at ages 9 and 11 as reported by the mother/chief carer.

Age (years)	Variable	Activity	Often	Sometimes	Not at all	NK ^a^	N
9	ku417	Prays	9.9%	31.9%	49.1%	9.1%	3929
11	kw4126	Prays	7.1%	24.0%	52.7%	16.2%	3683
9	ku416	Attends ^b^	15.1%	27.9%	57.0%	-	3910
11	kw4125	Attends ^b^	11.9%	22.3%	65.8%	-	3705

^a^ Not known; ^b^ Attends a place of worship (church, mosque, etc.)

**Table 3b.** Frequency that *girls* pray and/or attend a place of worship at ages 9 and 11 as reported by the mother/chief carer.

Age (years)	Variable	Activity	Often	Sometimes	Not at all	NK ^a^	N
9	ku417	Prays	12.7%	38.9%	40.0%	8.4%	3873
11	kw4126	Prays	9.1%	28.7%	45.2%	17.1%	3675
9	ku416	Attends ^b^	17.3%	31.4%	51.3%	-	3846
11	kw4125	Attends ^b^	15.1%	23.2%	61.7%	-	3704

^a^ Not known; ^b^ Attends a place of worship (church, mosque, etc.)

### Attendance at a place of worship

Although questionnaires were administrated annually so that changes in the frequency with which the child attended Sunday School was monitored from ages 4 to 11 years, there were only two occasions during this time frame that the frequency with which the child attended a place of worship was obtained ( Table 3). From the data collected there was a reduction in the proportion attending often or sometimes from 45.8% at age 9 to 36.3% at age 11. This drop was mirrored among the boys (43.0% at age 9 to 34.2 % at age 11) and girls (48.7% at age 9 to 38.3% at age 11), although at each age the girls were more likely to have attended than the boys ( Table 3a, Table 3b).

### Relevant interests of the child

The mother or chief carer of the child was asked on two occasions (when the child was aged 9 and at 11) how interested he/she was in: (a) the meaning of life and (b) religion. The replies are shown in Table 4. These indicate that whereas the interest in religion appeared to decrease between the two years, the interest in the meaning of life increased. The same trends were apparent among the boys and girls, but the girls were more likely to show interest in religion than the boys ( Table 4a, Table 4b).

**Table 4.** Amount of interest child takes in religion and in the meaning of life at ages 9 and 11 as reported by the mother/chief carer.

Age (years)	Interest in	Variable	Very interested	Somewhat interested	Not interested	Not sure	N
9	Religion	ku414	9.8%	52.1%	32.2%	5.9%	8040
11	Religion	kw4124	7.8%	44.6%	40.0%	7.5%	7239
9	Meaning of life	ku412	9.5%	44.1%	35.9%	10.4%	7962
11	Meaning of life	kw4122	10.6%	48.2%	30.3%	10.8%	7222

**Table 4a.** Amount of interest *boys* take in religion and in the meaning of life at ages 9 and 11 as reported by the mother/chief carer.

Age (years)	Interest in	Variable	Very interested	Somewhat interested	Not interested	Not sure	N
9	Religion	ku414	7.4%	48.5%	38.0%	6.2%	4047
11	Religion	kw4124	6.5%	41.0%	44.3%	8.2%	3619
9	Meaning of life	ku412	9.2%	43.4%	36.8%	10.7%	4013
11	Meaning of life	kw4122	10.4%	46.9%	31.9%	10.8%	3613

**Table 4b.** Amount of interest *girls* take in religion and in the meaning of life at ages 9 and 11 as reported by the mother/chief carer.

Age (years)	Interest in	Variable	Very interested	Somewhat interested	Not interested	Not sure	N
9	Religion	ku414	12.2%	55.7%	26.4%	5.7%	3987
11	Religion	kw4124	9.2%	48.3%	35.6%	6.9%	3612
9	Meaning of life	ku412	9.9%	44.8%	35.1%	10.2%	3943
11	Meaning of life	kw4122	10.8%	49.6%	28.7%	10.9%	3601

### Type of faith

There is, unfortunately, no direct question concerning the type of faith the child was brought up in. However, there are two ways in which this may be addressed, although neither is perfect:

a) When aged 27, the G1s were asked ‘what sort of faith would you say you have? The answers (YPG3040) indicated that 19.0% at this age claimed to be Church of England, 3.6% as Roman Catholic and 5.6% in other Christian denominations, but the vast majority stated that they had no faith. A follow-on question enquired whether they had been brought up in this faith (YPG3060): 16.0% said yes. Taking those who said ‘yes’ and cross-tabulating with YPG3040 indicated that 28.9% were probably brought up in the Church of England, 6.2% as Roman Catholics and 55.6% with no faith.
b) When the child was aged 9, each parent was asked what type of faith they had, and whether they were bringing the child up in that faith. As shown in Table 5, a high proportion of each of the parents stated that they were bringing the child up in their own faith – the mothers more so than the fathers. Calculation of the numbers of children brought up in the Church of England, Roman Catholic and no faith indicated 71.9% as Church of England, 7.9% as Roman Catholic and 5.2% as none.

**Table 5a.** The types of faith of the G0 parents when their son was aged 9, and whether the parents indicated that they were bringing their *boys* up in the same faith.

Type of Faith	G0 mother [P4044] ^a^	G0 father [PM4044] ^a^
Church of England	60.6 %	53.1 %
Roman Catholic	7.6 %	7.9 %
Other Christian	12.3 %	11.0 %
None	15.8 %	24.7 %
Total no. replying	3941	1796
Bringing child up in same faith	[P4048]	[PM4048]
- Yes	71.2 %	60.1 %

^a^Variable name

**Table 5b.** The types of faith of the G0 parents when their *girls* were aged 9, and whether the parent indicated that they were bringing them up in the same faith.

Type of Faith [variable]	G0 mother [P4044] ^a^	G0 father [PM4044] ^a^
Church of England	60.7 %	52.7 %
Roman Catholic	8.0 %	7.6 %
Other Christian	12.2 %	10.2 %
None	15.9 %	25.9 %
Total no. replying	3797	1728
Bringing child up in same faith	[P4048]	[PM4048]
- Yes	72.9 %	61.6 %

^a^Variable name

### Times when the child’s faith or belief changed

There were three questions asked when the G1 was aged 30 concerning changes to faith or belief. The age at which such changes occurred is given, thus allowing identification of children who had experienced such changes at various stages of their childhood. The changes noted were identified by the questions outlined in Table 6. Those participants who recorded having experienced such events were asked to describe them if they wished. Their answers are recorded as text and are available to bona fide researchers on application to the ALSPAC Executive.

**Table 6.** The proportion of G1 responders (aged 27+) answering questions on changes in belief/faith in their childhood. %s are of the total numbers of individuals answering the question.

	Variable	Male N(%)	Female N(%)	All N(%)
Had a religious or spiritual experience that changed life	YPG3181	62 (4.1%)	108 (3.7%)	170 (3.8%)
Had a significant gain in faith/belief	YPG3191	59 (3.9%)	126 (4.3%)	185 (4.1%)
Had a significant loss of faith/belief	YPG3201	145 (9.6%)	244 (8.2%)	389 (8.7%)

### Education in school

We have already shown in a previous Data Note [ Tohidinik *et al.*, 2025] that the data concerning the G1s in ALSPAC are linked to information on whether they had attended a Faith School during their childhood. Information available included number of years attending: (i) a faith primary school, and (ii) a faith secondary school ( Table 7). The majority of such schools attended by the G1s were Christian – either Church of England (Anglican) or Roman Catholic.

**Table 7.** Details available on Christian faith schools attended, extracted from the national pupil database (n = 12,825 children).

Question	Variable name
Ever attended a Christian faith school	faith_school_binary
No. years attended a Christian faith school	faith_school_yrs
Whether attended a Christian faith school at primary, secondary, or both levels	faith_school_level2
No. years attended a Christian faith school at primary level	primary_faith
No. years attended a Christian faith school at secondary level	secondary_faith
No. years attended CofE faith schools	Anglican
No. years attended Roman Catholic faith schools	Roman
Ever attended a CofE faith school	Anglican_cat
Ever attended a RC faith school	Roman_cat

CofE Church of England (Anglican)

In addition, ALSPAC carried out two surveys of primary schools in the Avon area attended by ALSPAC children. Questions asked of the class teacher when the children were in School Years 3 and 6 (approximate ages 8 and 11 years) concerned the length of time the class spent studying particular subjects. The numbers of complete hours per week dedicated to religious education (RE) are shown in Table 8, but any additional number of minutes per week is given by the accompanying b variable indicated.

**Table 8.** No. of hours spent each week in School Years 3 and 6 on religious education (state funded schools including faith and non-faith schools): data obtained from class teachers of ALSPAC children in the Avon area.

No. full hours/week ^a^	SY3 ^b^, N(%)	SY6 ^b^, N(%)
0	504 (40.4%)	564 (41.3%)
1	674 (54.0%)	724 (53.0%)
2+	70 (5.6%)	79 (5.7%)
Total	1248 (100%)	1367 (100%)
Variable	SB210a,b	SF210a,b

^a^ the additional number of minutes as well as hours are also available in variables SB210b and SF210b; ^b^ SY = school year: children in school years 3 and 6 were aged approximately 8 and 11 years.

### Other aspects of religion/faith relevant to the childhoods of the G1 cohort

There was one other detail that was relevant to the ways in which aspects of faith impacted on the developing child. When the child was 18 months of age the mother was asked: ‘Are there any foods which you do not allow him/her to eat?’ 59% replied positively [kd345]. For those who replied yes, mothers were asked to describe which foods (these answers were typed verbatim and are available for interested researchers); the mother was then asked for what reason: she was given six possible options, one of which was ‘religious/moral’ [kd351]. 4.1% of the G1 children were given this reason – unfortunately, the question was designed in such a way that it is not clear which foods were avoided for each of the different reasons, and consequently this question should be avoided.

## Conclusions

Although, in retrospect, the data concerning the child’s RSBB could have been improved, there is much available that provides useful insights into the religious exposures of the individuals during childhood. They indicate that girls are more exposed to, and more interested in, religion even at early ages. These data are available for investigations as to whether such childhood exposures have any impact on the individual as he/she heads into adulthood and beyond.

## Data Availability

ALSPAC data access is through a system of managed open access. The steps below highlight how to apply for access to the data included in this paper and all other ALSPAC data. Note that variable names are included in the Tables within this paper. Please read the ALSPAC access policy ( https://www.bristol.ac.uk/media-library/sites/alspac/documents/researchers/data-access/ALSPAC_Access_Policy.pdf) which describes the process of accessing the data and biological samples in detail, and outlines the costs associated with doing so. You may also find it useful to browse our fully searchable research proposals database ( https://proposals.epi.bristol.ac.uk/), which lists all research projects that have been approved since April 2011. Please submit your research proposal ( https://proposals.epi.bristol.ac.uk/) for consideration by the ALSPAC Executive Committee using the online process. You will receive a response within 10 working days to advise you whether your proposal has been approved. If you have any questions about accessing data, please email: alspac-data@bristol.ac.uk (data) or bbl-info@bristol.ac.uk (samples). The ALSPAC data management plan ( http://www.bristol.ac.uk/media-library/sites/alspac/documents/researchers/data-access/alspac-data-management-plan.pdf) describes in detail the policy regarding data sharing, which is through a system of managed open access.
