# Supplementary material for: Details of exposure during childhood of the original children (G1s) in the ALSPAC study to aspects of religious faith and spirituality
Source: Wellcome Open Res. 2026 May 20;11:88. Originally published 2026 Feb 3. [Version 2] doi: 10.12688/wellcomeopenres.25712.2 (PMC13221624; doi:10.12688/wellcomeopenres.25712.2)
Supplement: Peer review history

# Reviewer report: Reviewer response for version 2

**DOI:** 10.21956/wellcomeopenres.29219.r156042

**Published:** 2026-05-29

**Authors:** Howard Worsley

**Affiliations:** 1 Trinity College Bristol, Bristol, England, UK

I am happy with the responses.

Are sufficient details of methods and materials provided to allow replication by others?

Partly

Is the rationale for creating the dataset(s) clearly described?

Yes

Are the datasets clearly presented in a useable and accessible format?

Yes

Are the protocols appropriate and is the work technically sound?

Yes

Reviewer Expertise:

NA

I confirm that I have read this submission and believe that I have an appropriate level of expertise to confirm that it is of an acceptable scientific standard.

# Reviewer report: Reviewer response for version 2

**DOI:** 10.21956/wellcomeopenres.29219.r156043

**Published:** 2026-05-22

**Authors:** Dorota Weziak-Bialowolska

**Affiliations:** 1 Harvard University, Cambridge, USA; 2 Kozminski University (Ringgold ID: 49777), Warsaw, Masovian Voivodeship, Poland

I am satisfied with the revisions

Are sufficient details of methods and materials provided to allow replication by others?

Yes

Is the rationale for creating the dataset(s) clearly described?

Yes

Are the datasets clearly presented in a useable and accessible format?

Partly

Are the protocols appropriate and is the work technically sound?

Yes

Reviewer Expertise:

My research focuses on the psychological, social, and spiritual determinants of well-being across the life course, including childhood predictors of adult outcomes. I conduct cross-national longitudinal analyses, including work within the Global Flourishing Study, examining how early-life experiences relate to adult health and well-being, religiosity, and broader flourishing. My expertise includes outcome-wide and meta-analytic approaches to studying well-being across diverse cultural contexts.

I confirm that I have read this submission and believe that I have an appropriate level of expertise to confirm that it is of an acceptable scientific standard.

# Reviewer report: Reviewer response for version 1

**DOI:** 10.21956/wellcomeopenres.28310.r147452

**Published:** 2026-02-24

**Authors:** Dorota Weziak-Bialowolska

**Affiliations:** 1 Harvard University, Cambridge, USA; 2 Kozminski University (Ringgold ID: 49777), Warsaw, Masovian Voivodeship, Poland

This is a useful Data Note describing an important resource. I have a few comments that may help improve clarity and transparency.

 1. The opening statements require appropriate references. In particular: *“*

*There is increasing evidence that features of childhood are strongly related to adult mental health (reference needed), but exposures to religion or spirituality during childhood or adolescence are rarely considered (reference needed). Studies considering the positive or negative effects of religiosity have mainly started by obtaining information from adults, but few have looked at the longitudinal effects of exposure to aspects of RSBB during childhood (reference needed).”*

 These are important framing claims and should be supported with citations. Adding relevant references would strengthen the scientific grounding of the paper and better situate the dataset within the existing literature.

 The authors may wish to consult recent cross-national longitudinal work on childhood predictors (self-reported retrospectively) of adult religiosity and spirituality, including, for example:

 Reference 1

 Reference 2

 Reference 3

 Reference 4

 These are offered simply as potential starting points rather than as required citations.

 2. In Tables 2, 2a and 2b, the “not at all” and “< once per month” categories are combined. These represent meaningfully different levels of engagement and, if possible, should be presented separately. This would improve interpretability of the attendance distributions. It would also be helpful to report, for each variable, the number (n) and percentage (%) of missing observations (i.e., no response). Given that this is a Data Note, clear information about missingness is important for secondary users of the dataset.

 3. Figure 1 presents those attending at least “1–3 times per month,” but does not reflect those attending “< once per month,” who are still participating to some extent. Either the figure should include all attendance categories, or the caption should clearly specify that it refers only to attendance of at least 1–3 times per month. As currently presented, it may be misleading.

 4. The caption to Table 2c refers to “at least once a month,” whereas the original survey response option was “1–3 times per month.” Unless it is possible to distinguish those attending once per month from those attending 2–3 times per month, the caption should reflect the exact wording of the response category to avoid over-interpretation.

 5. More generally, please consider adding n and % of missing observations to each table. This would improve transparency and make the dataset easier to use analytically.

 Overall, the dataset is clearly of value. Addressing the points above would improve precision and usability without requiring substantive changes.

Are sufficient details of methods and materials provided to allow replication by others?

Yes

Is the rationale for creating the dataset(s) clearly described?

Yes

Are the datasets clearly presented in a useable and accessible format?

Partly

Are the protocols appropriate and is the work technically sound?

Yes

Reviewer Expertise:

My research focuses on the psychological, social, and spiritual determinants of well-being across the life course, including childhood predictors of adult outcomes. I conduct cross-national longitudinal analyses, including work within the Global Flourishing Study, examining how early-life experiences relate to adult health and well-being, religiosity, and broader flourishing. My expertise includes outcome-wide and meta-analytic approaches to studying well-being across diverse cultural contexts.

I confirm that I have read this submission and believe that I have an appropriate level of expertise to confirm that it is of an acceptable scientific standard.

## References

[1] : Childhood experiences and adult prayer or meditation in 22 countries around the world. *Scientific Reports* .2025;15(1) : 10.1038/s41598-025-99796-x 10.1038/s41598-025-99796-x 40307349 PMC12044052

[2] : Childhood predictors of belief in life after death across 22 countries. *Scientific Reports* .2025;15(1) : 10.1038/s41598-025-91615-7 10.1038/s41598-025-91615-7 40307319 PMC12043831

[3] : Childhood predictors of adults’ belief in god, gods, and spiritual forces across 22 countries. *Scientific Reports* .2025;15(1) : 10.1038/s41598-025-98796-1 10.1038/s41598-025-98796-1 40307380 PMC12044003

[4] : Childhood predictors of religious reading: a cross-national analysis in the Global Flourishing Study. *Scientific Reports* .2025;15(1) : 10.1038/s41598-025-10142-7 10.1038/s41598-025-10142-7 40640308 PMC12246113

# Response

**Published:** 2026-04-20

**Authors:** Yasmin Iles-Caven

**Affiliations:** Bristol Medical School: Population Health Sciences, University of Bristol, Bristol, UK

Thank you very much for your useful suggestions, particularly the references, which we have now incorporated into the Data Note

# Reviewer report: Reviewer response for version 1

**DOI:** 10.21956/wellcomeopenres.28310.r146822

**Published:** 2026-02-13

**Authors:** Howard Worsley

**Affiliations:** 1 Trinity College Bristol, Bristol, England, UK

**Details of exposure during childhood of the original children** **(G1s) in the ALSPAC study to aspects of religious faith and** **spirituality.**

 Typos

 -in abstract –‘Information collected from the study parents on their own religious…’ should be ‘Information collected from the study of parents on their own religious…’

 -in introduction-‘is much under-researched’ should be ‘is considerably under-researched’

 On a few occasions, the article identifies its omissions. This is helpful eg ‘Unfortunately, ALSPAC did not collect information on whether the child was being taught to pray during the early years’…or ‘There is, unfortunately, no direct question concerning the type of faith the child was brought up in.’. Maybe this could be identified in the introduction so that the reader knows what to expect.

 To improve the article and make it more appealing, I would like to ask the researchers to offer more in the conclusion which is extremely short. As well as noting the report’s inadequacies (ie  ‘in retrospect, the data concerning the child’s RSBB could have been improved,’ and saying that ‘there is much available that provides useful insights into the religious exposures of the individuals during childhood’, they should be more precise in saying;

1. What data could have been improved
2.  What useful insights into the religious exposures of the individuals during childhood  have been identified.

Are sufficient details of methods and materials provided to allow replication by others?

Partly

Is the rationale for creating the dataset(s) clearly described?

Yes

Are the datasets clearly presented in a useable and accessible format?

Yes

Are the protocols appropriate and is the work technically sound?

Yes

Reviewer Expertise:

Childhood studies, theology, qualitative research methodology

I confirm that I have read this submission and believe that I have an appropriate level of expertise to confirm that it is of an acceptable scientific standard, however I have significant reservations, as outlined above.

# Response

**Published:** 2026-04-20

**Authors:** Yasmin Iles-Caven

**Affiliations:** Bristol Medical School: Population Health Sciences, University of Bristol, Bristol, UK

Thank you very much for your very helpful comments

 Typos

 -in abstract –‘Information collected from the study parents on their own religious…’ should be ‘Information collected from the study of parents on their own religious…’

 -in introduction-‘is much under-researched’ should be ‘is considerably under-researched’ **Many thanks, these have been changed.**

 On a few occasions, the article identifies its omissions. This is helpful eg ‘Unfortunately, ALSPAC did not collect information on whether the child was being taught to pray during the early years’…or ‘There is, unfortunately, no direct question concerning the type of faith the child was brought up in.’. Maybe this could be identified in the introduction so that the reader knows what to expect. We have inserted a warning of these omissions in the Abstract as well as the Introduction

   To improve the article and make it more appealing, I would like to ask the researchers to offer more in the conclusion which is extremely short. As well as noting the report’s inadequacies (ie  ‘in retrospect, the data concerning the child’s RSBB could have been improved,’ and saying that ‘there is much available that provides useful insights into the religious exposures of the individuals during childhood’, they should be more precise in saying;

1. What data could have been improved
2.  What useful insights into the religious exposures of the individuals during childhood  have been identified.

**We have now described the data that could be improved in the Conclusion section, where we also describe the findings in regard to the sex of the child. We do not have further useful insights at the moment but hope that other researchers will be able to use the data to answer important questions concerning faith.**
